# Magic electron affection in preparation process of silicon nanocrystal

**DOI:** 10.1038/srep09932

**Published:** 2015-04-24

**Authors:** Wei-Qi Huang, Shi-Rong Liu, Zhong-Mei Huang, Ti-Ger Dong, Gang Wang, Cao-Jian Qin

**Affiliations:** 1 Institute of Nanophotonic Physics, Guizhou University, Guiyang 550025(China); 2 State Key Laboratory of Ore Deposit Geochemistry Institute of Geochemistry, Chinese Academy of Science Institute of Geochemistry, Guiyang 550003(China)

## Abstract

It is very interesting that magic electron affection promotes growth of nanocrystals due to nanoscale characteristics of electronic de Broglie wave which produces resonance to transfer energy to atoms. In our experiment, it was observed that silicon nanocrystals rapidly grow with irradiation of electron beam on amorphous silicon film prepared by pulsed laser deposition (PLD), and silicon nanocrystals almost occur in sphere shape on smaller nanocrystals with less irradiation time of electron beam. In the process, it was investigated that condensed structures of silicon nanocrystals are changed with different impurity atoms in silicon film, in which localized states emission was observed. Through electron beam irradiation for 15min on amorphous Si film doped with oxygen impurity atoms by PLD process, enhanced photoluminescence emission peaks are observed in visible light. And electroluminescence emission is manipulated into the optical communication window on the bigger Si-Yb-Er nanocrystals after irradiation of electron beam for 30min.

Silicon is the most important semiconductor material for the electronic industries. But the optical properties of silicon are relatively poor, owing to its indirect band gap which precludes the efficient emission and absorption of light. Due to the modification of the energy structure afforded by quantization, silicon nanocrystals emerge as ideal candidates for photonic applications involving efficient radiation recombination. The emission from silicon nanocrystals has characteristic features: photoluminescence (PL) intensity increases and its wavelength occurs blue-shift with decreasing crystal size. Further, localized states due to impurity atoms, such as oxygen or nitrogen, and surface or interface defects have been suggested that leads to a stabilization of the PL wavelength for smaller silicon nanocrystals^1^,^2^,^3^.

Silicon nanocrystals have been studied intensively over the past decade^4^,^5^. The popular methods for fabricating silicon nanocrystals are self-assembly from silicon-rich silicon oxide matrices^6^,^7^ and plasma synthesis^8^,^9^,^10^,^11^. The interesting method to fabricate silicon nanocrystals is growth under photons interaction^12^,^13^,^14^. In the first case, SiO*x* (with *x* < 2) is formed by a thin-film deposition technique such as pulsed laser deposition (PLD). Subsequent high-temperature annealing of the substoichimetric film (typically 900~1100°C) produces a phase separation between Si and SiO_2_ with the formation of Si nanoclusters. The dimensions, crystallinity and size distribution of the nanoclusters depend on the Si excess, the temperature and the annealing time^5^,^6^.

In the article, the most interesting and simplest method discovered in our experiment for fabricating silicon nanocrystals is self-assembly growth by assistance of electron interaction, in which silicon nanocrystals rapidly grow with irradiation of electron beam on amorphous silicon film prepared by PLD, and shape of silicon nanocrystals is almost sphere when crystal size is smaller with less irradiation time of electron beam. The method of electron affection could be used to replace the traditional annealing methods in preparing process of silicon nanocrystals. In the process, it was investigated that condensed structures of silicon nanocrystals are changed with different impurity atoms in silicon film, for examples oxygen or Er atoms make a stronger condensed affection than doing of nitrogen or Yb atoms in impurity, in which various localized states emission was measured.

It is very interesting that magic electron affection promotes the growth of nanocrystals, whose physical mechanism may be from nanoscale characteristics of electronic de Broglie wave which produces resonance to transfer energy to crystal atoms.

In natural sciences, many analogous structures and properties occur on different size hierarchy, for example in nanoscale space related to electronic de Broglie wavelength and in sub-micrometer scale related to photonic de Broglie wavelength, in which the photon affection from nanosecond or femtosecond laser is used to fabricate periodic surface structures with 100nm spatial periods on silicon^15^,^16^, and the electron affection is used to prepare silicon nanocrystals, which were demonstrated in experiment.

The amorphous silicon film was prepared in the combination fabrication system with pulsed laser etching (PLE) and PLD devices (see Methods), as shown in Fig. 1. Then, the amorphous silicon film was exposed under electron beam with 0.5 nA/nm^2^ for 5~30min in Tecnai G2 F20 system, it was observed that silicon nanocrystals rapidly grow with irradiation of electron beam and various shape of crystals forms with different irradiation time on the amorphous silicon film with impurity (see Methods). In Fig. 2(a), the image of transmission electron microscope (TEM) shows Si quantum dots (QDs) embedded in the Si_y_N_x_ amorphous film prepared in PLD device with nitrogen gas tube, whose diameter is about 2~5nm after irradiation under electron beam for 15min. The Si–Yb QDs structures are built after irradiation under electron beam for 20min on the amorphous film with impurity prepared in PLD device with Si and Yb bars, as shown in the TEM image of Fig. 2(b).

It is interesting that gradually growing process of QDs structure was observed under electron beam with increase of irradiation time. For example, not only QD structure has been observed after irradiation for 5min (TEM image in Fig. 3(a)), and a few QDs are observed after irradiation for 15min under electron beam (TEM image in Fig. 3(b)) on amorphous Si film prepared in oxygen gas. Figure 3(c) shows the Fourier transform image on the structures with QDs embedded in SiO_x_. As shown in TEM images of Fig. 4, no QD structure occurs after electron beam irradiation for 5min (a), a few QDs appear after radiation for 20min (b) and QDs structure has been broken over irradiation time of 30min (c) on the Si–Yb amorphous film prepared by PLD process in the device related to Fig. 1(b). The inset in Fig. 4(a) shows composition of Si and Yb in X-ray energy spectrum on the amorphous film.

In the crystallizing process under irradiation of electron beam, it was observed that condensed speed is different to form different structures of silicon nanocrystals with different impurity atoms on silicon film, for examples oxygen or Er atoms make a stronger condensed affection than doing of nitrogen or Yb atoms in impurity. In Fig. 5, TEM images show different silicon nanocrystals with different impurity gas atoms after irradiation of electron beam for 30min, such as in nitrogen gas (a), in oxygen gas (b) and in SF gas (c) on the samples prepared in the device related to Fig. 1(a), in which the film structure of Si–N nanocrystals is still kept, but the film structures of Si–O and Si–S nanocrystals have been broken. As shown in Fig. 6, TEM images show different silicon nanocrystals with different impurity solid atoms, such as Yb bar (a), Ge bar (b) and Er bar (c) on the samples prepared in the device related to Fig. 1(b), in which the film structure of Si–Yb nanocrystals is still kept, but the film structures of Si–Ge and Si–Er nanocrystals have been broken. Here, it is obvious that some atoms have a stronger condensed ability, such as Er atom.

On the silicon nanocrystal samples prepared by using irradiation of electron beam in the PLD device with oxygen gas tube related to Fig. 1(a), photoluminescence (PL) spectra were investigated in various impurity atoms after annealing for different time (see Methods). Figure 7(a) shows silicon QDs embedded in SiO_x_ prepared by using irradiation of electron beam for 15min, whose sharper PL peak at 604nm is observed after annealing at 1050° C for 20min as shown in Fig. 7(b). Various PL spectra are observed with annealing time of 10min, 15min or 20min on the samples, as shown in Fig. 7(c), which shows that annealing time of 20min is suitable for localized states emission.

In Fig. 8(a), Si QDs embedded in Si_y_N_x_ is observed on the sample prepared by using radiation of electron beam for 15 min in the device with nitrogen tube related to Fig. 1(a). PL peak at 605nm (near 2eV) on the sample is found in Fig. 8(b).

In the PLD device with Yb bar related to Fig. 1(b), Si–Yb QDs embedded in Si_y_N_x_ are prepared by using irradiation of electron beam for 15min, as shown in Fig. 9(a), whose PL peaks occur in Fig. 9(b). It is very interesting that in the PLD device with Yb and Er bars related to Fig. 1(b), bigger Si–Yb–Er nanocrystals with various shapes appear after irradiation of electron beam for 30min, as shown in Fig. 10(a), whose electroluminescence (EL) spectra occur in optical communication window in Fig. 10(b) (see Methods), which have the characteristics of localized states emission on surface defects of Si crystals. Here, the QC effect disappears and the localized states emission from defects and impurity of bigger nanocrystals surface or interface plays a main role.

In experiments, PL bands in visible light are observed on smaller Si nanocrystals such as Si QDs structures prepared by using irradiation of electron beam for less time, and localized states emission peaks are measured at some fixative wavelengths with different impurity atoms bonding on QDs surface. In Fig. 11, simulation analysis of PL spectra shows that PL band emission (Fit peak 3) on Si QDs with various sizes belongs to the QC effect emission, and localized states emission (Fit peaks 1 and 2) comes from impurity atoms bonding on nanocrystals surface, which form competition in physical mechanism.

A physical model of the localized states emission could be built on Si nanocrystals doped with various impurity atoms by using irradiation of electron beam, in which electron from bottom pumping states of conduction band (CB) opened, owing to quantum confinement (QC) effect in Si QDs, is relaxed into the localized states due to impurity atoms bonding on QDs surface, and then enhanced PL emission is obtained due to recombination between electron in the localized states near CB and hole in the localized states near valence band. As shown in Fig. 12, electrons in pumping states of CB opened due to smaller Si QDs (<3nm) are relaxed into the localized states of Si = O bonds on QDs surface to form inverse population between up localized states and low localized states, then stimulated emission near 700nm could be built. In same way, the localized states due to Si – O – Si bonds on surface of smaller Si QDs (<2.5nm) could produce stimulated emission near 600nm. But the localized states due to Si–Yb – Er bonding on surface of bigger nanocrystals prepared by electron beam irradiation for longer time will be deeper position in band gap to manipulate emission wavelength into optical communication window. In fact, this physical model involves a four-level system for emission.

Figure 13 shows the cavity affection for stimulated emission on Si QDs embedded in SiO_x_ prepared by using irradiation of electron beam for 15min, in which the Purcell cavity (Q factor: ~2000) is fabricated by PLE process in the device related to Fig. 1.

In conclusion, various silicon nanocrystals are fabricated by using irradiation of electron beam on Si film prepared by PLD process, in which Si QDs with 2~5nm diameter could be obtained by controlling irradiation time of electron beam. Through electron beam irradiation for 15min and annealing at 1050°C for 20min on amorphous Si film doped with different impurity atoms by PLD process, enhanced PL emission peaks are observed in visible light. And EL emission wavelength could be manipulated into optical communication window by irradiation of electron beam for longer time to form bigger Si crystals doped with Yb and Er impurity atoms. In the process, physical phenomena and effects are very interesting, and a new way will be developed for fabrication of silicon nanostructures, which would have good application in emission materials and LED devices.

## Methods

### Preparation of amorphous silicon film

**Figure 1:**
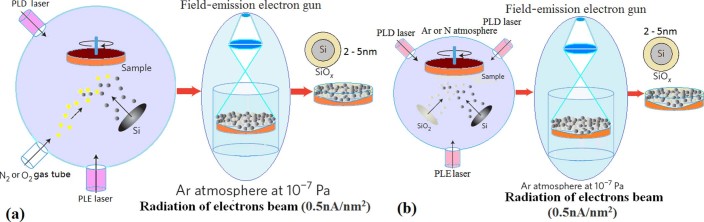
Combination fabrication system with electron beam irradiation device, PLD and PLE devices. (a) PLD impurity process through gas tube, in which at first the amorphous Si film forms, then Si nanocrystals grow up under irradiation of electron beam and Si QDs embedded in SiO_x_ or in Si_y_N_x_ are produced. (b) Impurity process by using PLD bars, such as SiO_2_, Ge, Yb or Er bar, in which Si–Ge QDs and Si–Yb–Er QDs embedded in SiO_x_ or in Si_y_N_x_ are produced under irradiation of electron beam.

Some silicon wafers of P-type (100) oriented substrate with 1–10 Ωcm were taken on the sample stage in the combination fabrication system with pulsed laser etching (PLE) and pulsed laser deposition (PLD) devices, as shown in Fig. 1. A pulsed Nd:YAG laser (wavelength: 1064nm, pulse length: 60 ns FWHM, repetition rate: 1000) was used to etch the Purcell micro-cavity on Si sample in PLE process. In the cavity, a third harmonic of pulsed Nd:YAG laser at 355nm was used to deposit the amorphous silicon film in PLD process. Figure 1(a) shows the impurity process on the amorphous silicon film through gas tube, such as nitrogen or oxygen gas. Figure 1(b) shows the impurity process on the amorphous silicon film by using PLD bars, such as SiO_2_, Ge, Yb or Er bar.

### Fabrication of silicon quantum dots under irradiation of electron beam

The amorphous silicon film was exposed under electron beam with 0.5 nA/nm^2^ for 5~30min in Tecnai G2 F20 system, in which electron beam from field‐emission electron gun, accelerated by 200 KV, has higher energy and better coherent. After irradiation under electron beam for 15min, silicon quantum dots (Si QDs) structures are built and embedded in SiO_x _(with *x* < 2) or Si_y_N_x_ (with *x* < 4 and y > 3) amorphous film related to oxygen or nitrogen gas tube respectively in the PLD device.

### Transmission electron microscope (TEM) analysis

In the TEM (JEM-2000FX) image, the silicon quantum dots and the silicon nanostructures were observed in the silicon amorphous films with impurity, and the compositions were measured on the samples by using analysis in X-ray energy spectra.

### Photoluminescence (PL) measurement

PL spectra of the samples were measured under the 514nm excitation by using RENISHAW Micro-Raman Systems at room temperature.

### Electroluminescence (EL) measurement

EL spectra were measured on the sample whose surface was deposited the ITO film for positive pole and bottom side was deposited the Au film for negative pole.

### Annealing process

The samples were sent into the annealing furnace filled with nitrogen atmosphere to make annealing at 1050°C for 10min, 15min or 20min. The PL spectra show that annealing time of 20min is suitable for localized states emission.

## Author Contributions

H.W.Q. and L.S.R. conceived and designed the experiments. H.W.Q., H.Z.M., D.T.G. and W.G. analyzed the data. H.W.Q., L.S.R., H.Z.M., D.T.G., W.G. and Q.C.J. fabricated Si nanocrystals and measured PL spectra. H.W.Q., L.S.R., H.Z.M., D.T.G., W.G. and Q.C.J. performed the experiments. H.W.Q., H.Z.M., D.T.G. and W.G. wrote the manuscript. All authors reviewed the manuscript.

## Figures and Tables

**Figure 2:**
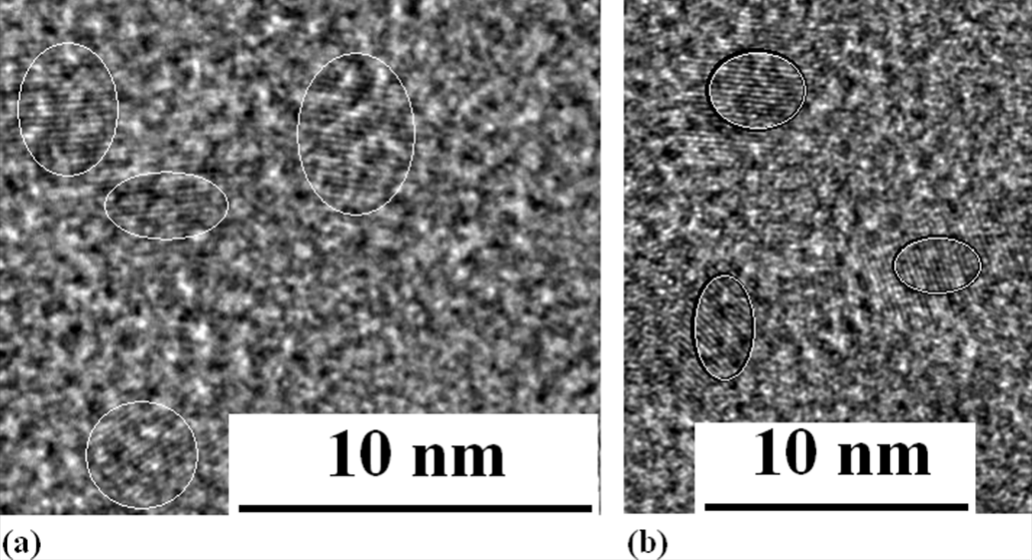
TEM images of QDs. (a) TEM image of Si QDs embedded in Si_y_N_x_ after irradiation under electron beam for 15min on amorphous Si film prepared in nitrogen gas. (b) TEM image of Si–Yb QDs embedded in Si_y_N_x_ after irradiation under electron beam for 20min.

**Figure 3:**
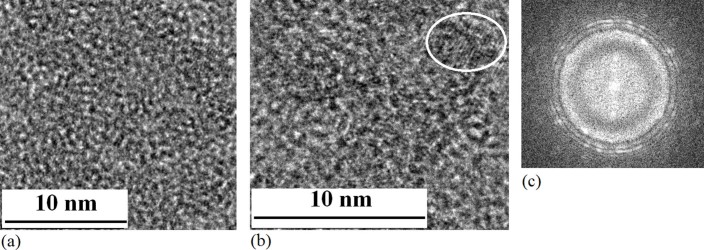
TEM images of Si QDs growing process. (a)TEM image of Si–O structure on amorphous Si film after irradiation of electron beam for 5min, in which not only QD structure has been observed. (b)TEM image of Si QDs embedded in SiO_x_, which are fabricated under electron beam irradiation on amorphous Si film prepared in oxygen gas. (c) Fourier transform image on the structures with QDs related to (b).

**Figure 4:**
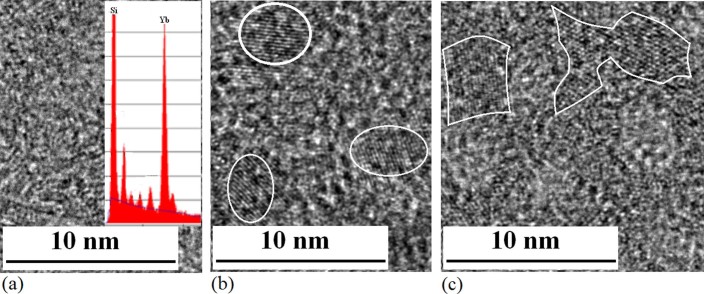
TEM images of Si nanocrystals growing process. (a) TEM image of Si–Yb–N structure on amorphous film after irradiation of electron beam for 5min, in which not only QD structure has been observed, and inset shows composition of Si and Yb in X-ray energy spectrum on the amorphous film. (b) TEM image of Si–Yb QDs embedded in Si_y_N_x_ amorphous film after irradiation of electron beam for 20min. (c) TEM image of Si–Yb nanocrystals with various shapes embedded in Si_y_N_x_ amorphous film after irradiation of electron beam over 30min, in which QDs structure has been broken

**Figure 5:**
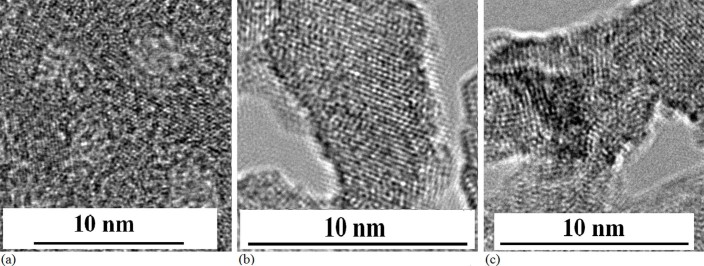
TEM images of different silicon nanocrystals prepared in different impurity gas atoms after irradiation of electron beam for 30min. (a) TEM image of Si–N nanocrystals with various shapes in amorphous film after irradiation of electron beam for 30min, in which the film structure is still kept. (b) TEM image of Si–O nanocrystals with various shapes in amorphous film after irradiation of electron beam for 30min, in which the film structure has been broken because of stronger condensed ability of O atoms. (c) TEM image of Si–S nanocrystals with various shapes in amorphous film after irradiation of electron beam for 30min, in which the film structure has been broken because of stronger condensed ability of S atoms.

**Figure 6:**
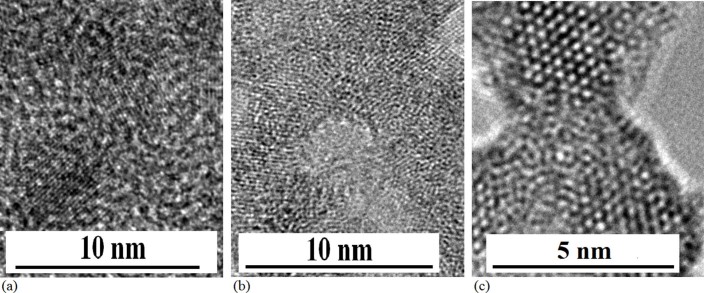
TEM images of different silicon nanocrystals prepared in different impurity solid atoms after irradiation of electron beam for 30min. (a)TEM image of Si–Yb nanocrystals with various shapes in amorphous film after irradiation of electron beam for 30min, in which the film structure is still kept. (b)TEM image of Si–Ge nanocrystals with various shapes in amorphous film after irradiation of electron beam for 30min, in which the film structure has been broken due to stronger condensed ability of Ge atoms. (c)TEM image of Si–Er nanocrystals with various shapes in amorphous film after irradiation of electron beam for 30min, in which the film structure has been broken because of stronger condensed ability of Er atoms.

**Figure 7:**
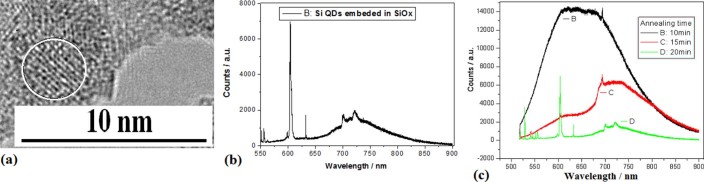
TEM image of Si QDs embedded in SiO_x_ and their PL spectra. (a)TEM image of silicon QDs embedded in SiO_x_ prepared by using irradiation of electron beam for 15min. (b)PL peaks on the Si QDs sample prepared with irradiation of electron beam for 15min and annealing at 1050°C for 20min. (c)Various PL spectra on the Si QDs samples prepared with irradiation of electron beam for 15min and annealing at 1050°C for 10min, 15min or 20min.

**Figure 8:**
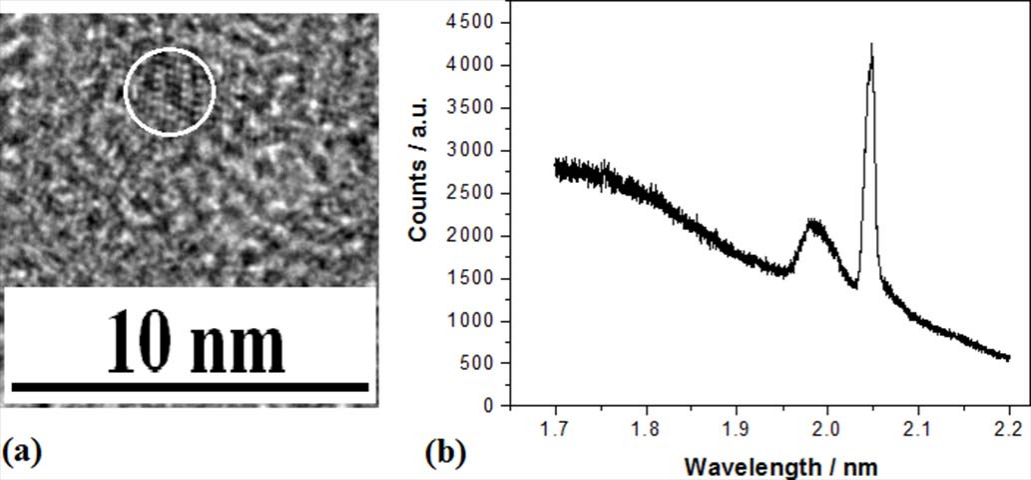
TEM image of Si QDs embedded in SiyNx and their PL spectra. (a) TEM image of silicon QDs embedded in Si_y_N_x_ prepared by using irradiation of electron beam for 15min. (b)PL peak near 2 eV on the Si QDs sample prepared in nitrogen with irradiation of electron beam for 15min and annealing at 1050°C for 20min.

**Figure 9:**
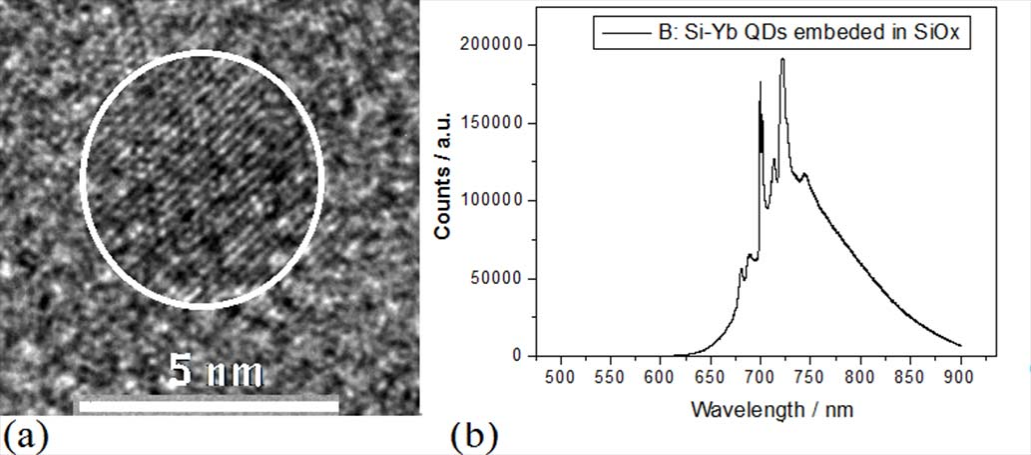
TEM image of Si–Yb QDs embedded in SiyNx and their PL spectra. (a) TEM image of Si–Yb QDs embedded in Si_y_N_x_ prepared by using irradiation of electron beam for 15min. (b) PL peak near 700nm on the Si-Yb QDs sample prepared in nitrogen with irradiation of electron beam for 15min and annealing at 1050°C for 20min.

**Figure 10:**
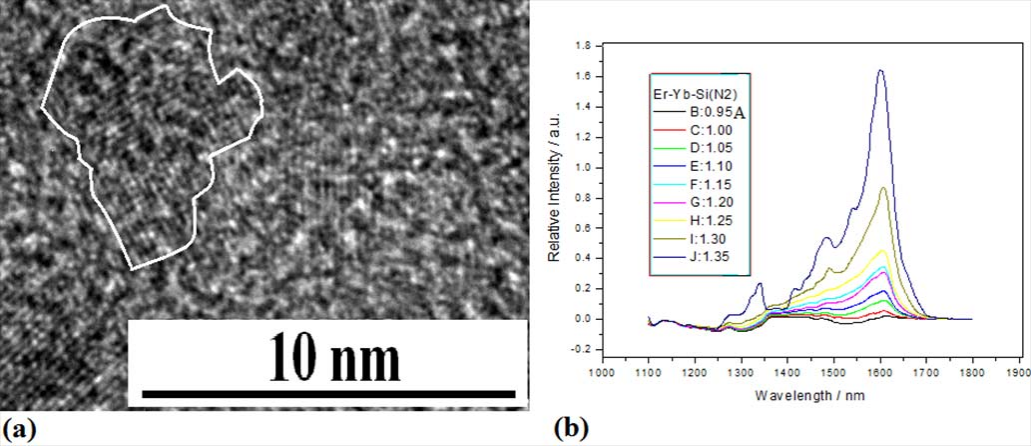
TEM image of Si -Yb-Er QDs embedded in SiyNx and their EL spectra. (b) TEM image of bigger Si–Yb–Er nanocrystals with various shapes embedded in Si_y_N_x_ prepared by using irradiation of electron beam for 30min. (b) EL emission in optical communication window on the Si–Yb–Er nanocrystals sample prepared in nitrogen with irradiation of electron beam for 30min and annealing at 1050°C for 20min, in which stimulated emission characteristics are observed near 1600nm.

**Figure 11:**
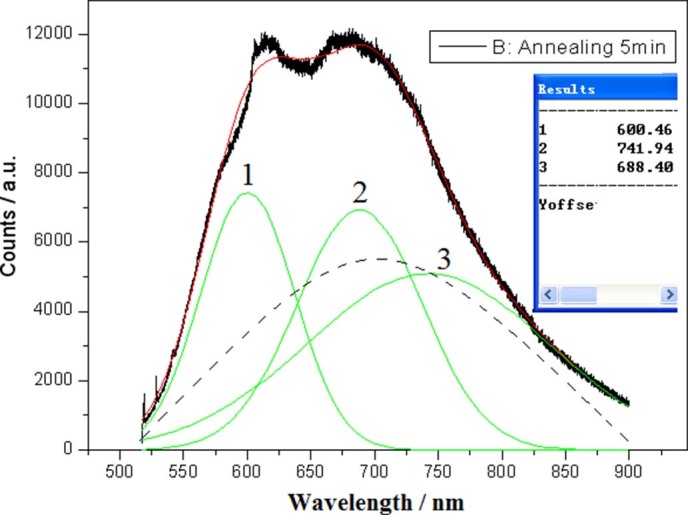
PL spectrum on Si QDs embedded in SiO_x_ prepared by using irradiation of electron beam for 10min, in which PL band emission (Fit peak 3) in Si QDs with various sizes belongs to the QC effect emission and localized states emission (Fit peaks 1 and 2) comes from impurity atoms bonding on nanocrystal surface, dash-line peak would describe pure QC effect emission if localized states disappeared.

**Figure 12:**
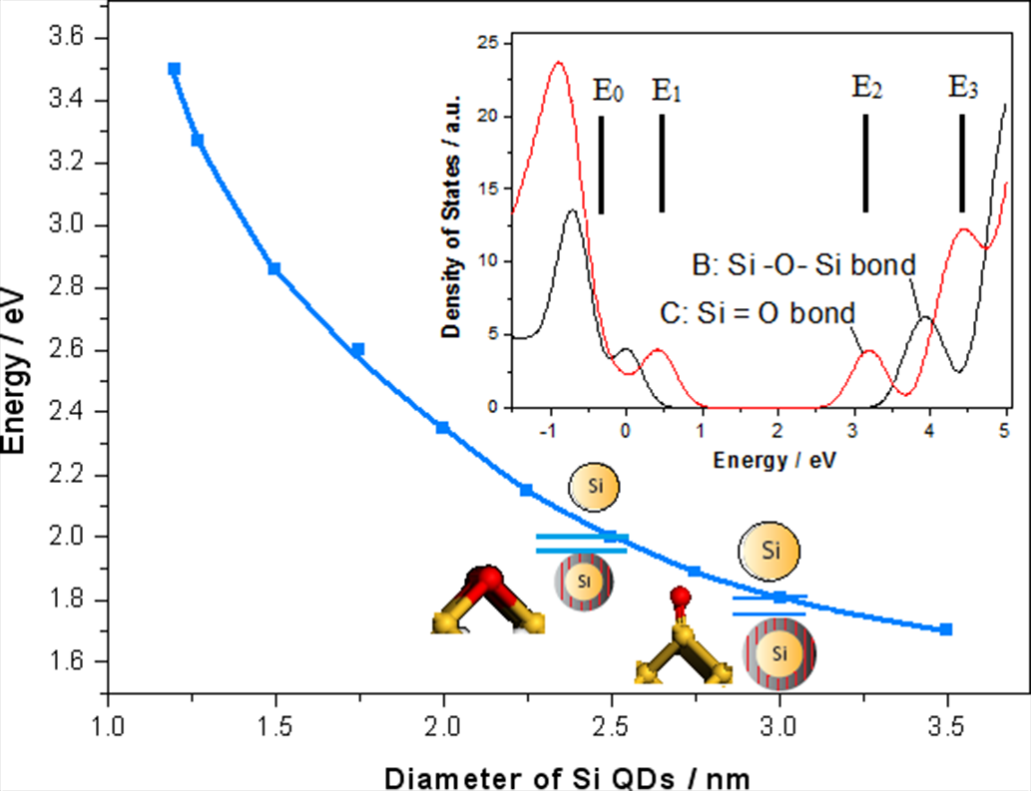
Physical model in Localized states emission, in which electrons in pumping states of CB opened due to Si QDs whose size is smaller than 3nm are relaxed into the localized states of Si = O bonds on QDs surface to form inverse population between up localized states and low localized states, then stimulated emission near 700nm is built.

**Figure 13:**
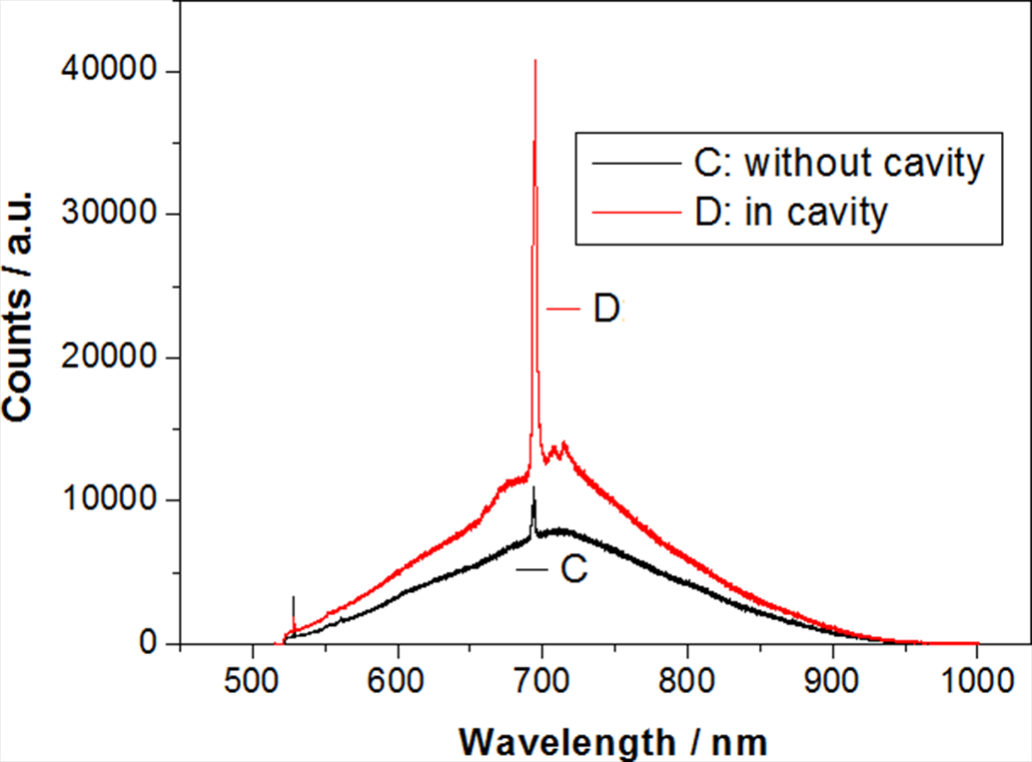
Cavity affection for stimulated emission on Si QDs embedded in SiO_x_ prepared by using irradiation of electron beam for 15min, in which the Purcell cavity (Q factor: ~2000) is fabricated by PLE process in the device related to Fig. 1.
